# Cholesterol Content Regulates the Interaction of αA-, αB-, and α-Crystallin with the Model of Human Lens-Lipid Membranes

**DOI:** 10.3390/ijms25031923

**Published:** 2024-02-05

**Authors:** Raju Timsina, Preston Hazen, Geraline Trossi-Torres, Nawal K. Khadka, Navdeep Kalkat, Laxman Mainali

**Affiliations:** 1 Department of Physics, Boise State University, Boise, ID 83725, USA; timsinaraju47@gmail.com (R.T.); nawalkhadka@boisestate.edu (N.K.K.); 2 Biomolecular Sciences Graduate Programs, Boise State University, Boise, ID 83725, USA; prestonhazen@u.boisestate.edu (P.H.); geralinetrossito@u.boisestate.edu (G.T.-T.); navdeepkalkat@u.boisestate.edu (N.K.)

**Keywords:** α-crystallin, αA-crystallin, αB-crystallin, percentage of membrane surface occupied (MSO), maximum percentage of membrane surface occupied (MMSO), binding affinity (K_a_), mobility parameter, maximum splitting, hydrophobicity, cholesterol, cholesterol bilayer domains, EPR spin-labeling method, cataracts

## Abstract

α-Crystallin (αABc) is a major protein comprised of αA-crystallin (αAc) and αB-crystallin (αBc) that is found in the human eye lens and works as a molecular chaperone by preventing the aggregation of proteins and providing tolerance to stress. However, with age and cataract formation, the concentration of αABc in the eye lens cytoplasm decreases, with a corresponding increase in the membrane-bound αABc. This study uses the electron paramagnetic resonance (EPR) spin-labeling method to investigate the role of cholesterol (Chol) and Chol bilayer domains (CBDs) in the binding of αAc, αBc, and αABc to the Chol/model of human lens-lipid (Chol/MHLL) membranes. The maximum percentage of membrane surface occupied (MMSO) by αAc, αBc, and αABc to Chol/MHLL membranes at a mixing ratio of 0 followed the trends: MMSO (αAc) > MMSO (αBc) ≈ MMSO (αABc), indicating that a higher amount of αAc binds to these membranes compared to αBc and αABc. However, with an increase in the Chol concentration in the Chol/MHLL membranes, the MMSO by αAc, αBc, and αABc decreases until it is completely diminished at a mixing ratio of 1.5. The K_a_ of αAc, αBc, and αABc to Chol/MHLL membranes at a mixing ratio of 0 followed the trend: K_a_ (αBc) ≈ K_a_ (αABc) > K_a_ (αAc), but it was close to zero with the diminished binding at a Chol/MHLL mixing ratio of 1.5. The mobility near the membrane headgroup regions decreased with αAc, αBc, and αABc binding, and the Chol antagonized the capacity of the αAc, αBc, and αABc to decrease mobility near the headgroup regions. No significant change in membrane order near the headgroup regions was observed, with an increase in αAc, αBc, and αABc concentrations. Our results show that αAc, αBc, and αABc bind differently with Chol/MHLL membranes at mixing ratios of 0 and 0.5, decreasing the mobility and increasing hydrophobicity near the membrane headgroup region, likely forming the hydrophobic barrier for the passage of polar and ionic molecules, including antioxidants (glutathione), creating an oxidative environment inside the lens, leading to the development of cataracts. However, all binding was completely diminished at a mixing ratio of 1.5, indicating that high Chol and CBDs inhibit the binding of αAc, αBc, and αABc to membranes, preventing the formation of hydrophobic barriers and likely protecting against cataract formation.

## 1. Introduction

Cataracts are the primary source of worldwide blindness today [1,2] and are believed to be a multifactorial process involving genetic and environmental factors that can be influenced by age [3,4], diabetes [5,6,7,8,9,10,11,12], corticosteroid use [13,14,15,16], vitrectomy [17,18], and hyperbaric oxygen treatment [19,20,21]. It is commonly believed that cataract formation predominantly results from oxidative stress that disrupts the membrane structure of the fiber cells comprising the eye lens, disrupting intrinsic protein function, promoting the aggregation of crystallins, and consequentially causing a buildup of insoluble high-molecular-weight (HMW) protein aggregates within the lens [3,21,22,23]. These HMW proteins are primarily developed from the aggregation of crystallin proteins (α-, β-, and γ-crystallin), which account for nearly 90% of the water-soluble lens proteins, with α-crystallin (αABc) accounting for about 40% of the total lens water-soluble proteins [24,25,26,27,28,29,30,31]. While the crystallins are normally soluble cytoplasmic proteins [32], previous studies have found that their membrane binding may cause their insolubilization, with nearly all water-insoluble lens crystallins being membrane-bound [33,34,35,36,37]. In addition to being the predominant lens protein, αABc has been shown to have the strongest affinity for the lens membrane [23,26,38,39,40,41,42,43,44] and becomes increasingly membrane-bound with cataract progression [23,34,38,39,45,46,47,48]. αABc exists in polydisperse oligomeric complexes comprised of αA-crystallin (αAc) and αB-crystallin (αBc) in a roughly 3:1 ratio of αAc to αBc. These oligomeric complexes have been shown to range from 10 to 40 subunits in size [49,50], with oligomers built of 24–28 subunits being the most commonly detected structures [50,51]. αAc and αABc have been shown to have a relatively high level of polydispersity [37], while αBc subunits have relatively less polydispersity and primarily form 24-mers [52,53]. αABc is a heteropolymer that functions as a molecular chaperone by preventing protein aggregation and providing tolerance to stress in the eye lens [23,29,31,54,55]. Additionally, αAc is primarily expressed in the eye lens, where it has protective effects [56,57], while αBc is found in several tissues, including the heart, kidneys, brain, and skeletal muscle, where it is believed to have a significant role in the protection and stabilization of cytoskeleton filaments [54,58,59,60]. Therefore, while αAc and αBc have apparent differences in structure, function, and use throughout the body, the exact role and interactions of each subunit remain unclear.

Prior studies have shown that αABc can interact with the lipid membranes [61,62] via hydrophobic interactions, and these interactions are predominantly modulated by the proteins’ surface hydrophobicity [26,41,42,45,48,63,64,65]. Moreover, using fluorescence, Su et al. investigated the binding of human αAc and αBc to bovine cortical membranes (CM) and human CM and nuclear membranes (NM) containing intrinsic membrane proteins and suggested that the mechanisms by which αAc and αBc interact with the fiber cell plasma membrane are different, with αAc exclusively interacting with lipids in the membrane [46]. Along this line of research, De Maio et al. showed that αBc differentially interacts with membranes made of different lipid headgroups [66]. In agreeance, past studies on synthetic lens membranes [64,67,68] and bovine lens-lipid membranes [43,45,69] showed that αABc can bind to the lens membrane, and this interaction reduces the mobility of the membrane near the headgroup region. In addition to high crystallin content in the lens, the eye lens fiber-cell plasma membrane has a uniquely high cholesterol (Chol) content [70,71,72,73,74], with the Chol/lipid molar ratio in the human eye lens membrane increasing with age [70,71,72]. With an increase in Chol concentration, Chol saturates the membrane and forms the lipid cholesterol domain (LCD) [71,75,76], and with a further increase in the Chol concentration, pure cholesterol bilayer domains (CBDs) start to form within the LCD [71,75,76]. Relatedly, prior in vivo studies have found that the knockout of Chol production in the lens results in the development of cataracts [77]. In previous fluorescent studies, Petrash et al. found that Chol does not significantly affect the binding of αAc, αBc, and αABc to lipid vesicles [48]. However, Tang et al. reported that Chol significantly decreases αABc binding to lipid vesicles. Therefore, how human αAc, αBc, and αABc bind with the lens membrane, the role of Chol and CBDs, and the physical properties (mobility, order, and hydrophobicity) of membranes with αAc, αBc, and αABc binding remain unclear.

Our earlier studies showed that a high Chol content leads to the formation of CBDs in the lens membrane [71,75,76,78]. Relatedly, we have detected the presence of CBDs at high Chol content in model membranes [79,80,81], lens-lipid membranes [71,75,76], and in the intact CM and NM isolated from human donors who were 40, 46, and 53 years old [78]. Our previous studies [82] and ongoing studies [23,63,67,68] showed that Chol and CBDs have positive physiological functions in maintaining the lens membrane and cytoplasm homeostasis, which in turn helps maintain lens transparency. Additionally, we have previously used electron paramagnetic resonance (EPR) spin-labeling methods to study the role of membrane lipids (phospholipids (PLs) and sphingolipids), Chol, and CBDs on the binding of native bovine lens αABc to the eye lens membrane [23,63,64,67,68]. From these studies, we have found that the lipid structure (acyl chain length, degree of acyl chain unsaturation, lipid headgroups, and lipid curvature) and the membrane lipid and Chol composition strongly modulate αABc membrane binding, and such binding significantly alters the physical properties of the membranes [63,64,67,68]. αABc membrane binding also increases membrane hydrophobicity on the surface, and when αABc binding is significantly reduced with the addition of Chol, there is a corresponding decrease in membrane surface hydrophobicity, indicating that αABc binds to the membrane via hydrophobic interactions and forms a hydrophobic barrier for the passage of polar molecules, supporting the barrier hypothesis in cataract development [67,68]. In this study, we used the EPR approach and investigated the association of recombinant human αAc, αBc, and reconstituted 3:1 heteromeric complex of αAc to αBc (i.e., αABc) to a Chol/model of human lens-lipid (Chol/MHLL) membranes with an increasing Chol concentration. The study in this manuscript provides new information in the field of αABc–membrane interactions, which includes understanding interactions of human αAc, αBc, and αABc with the Chol- and CBD-containing membranes, how these interactions affect the physical properties (mobility, order, and hydrophobicity) of membranes, and the inhibitory role of Chol and CBDs on αAc, αBc, and αABc binding to the membranes.

## 2. Results

### 2.1. Binding of Recombinant Human αAc, αBc, and αABc to Chol/MHLL Membranes

**Figure 1:**
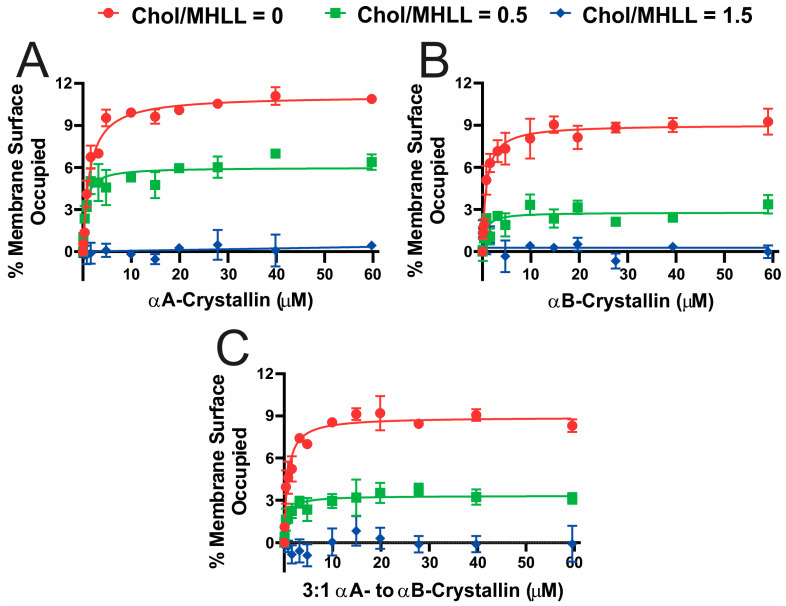
(**A**–**C**) The percentage of membrane surface occupied (MSO) plotted as a function of αA-crystallin (αAc), αB-crystallin (αBc), and 3:1 αA- to αB-crystallin (αABc) concentrations, respectively, for Chol/MHLL membranes. The Chol/MHLL mixing ratios are 0 (0 mol% Chol), 0.5 (33 mol% Chol), and 1.5 (60 mol% Chol). The concentration of lipids plus Chol was maintained at 11.4 mM, and αAc, αBc, and αABc were varied from 0 to ~60 μM. The mixed αAc, αBc, or αABc, and membrane samples were incubated at 37 °C for 16 h, and EPR measurements were taken at 37 °C. The data points in (**A**–**C**) were fitted with a one-site ligand-binding model in GraphPad Prism version 8.4.3 (San Diego, CA, USA) to estimate the K_a_. The error bars were calculated from the average of three independent experiments.

Figure 1A–C display the percentage of membrane surface occupied (MSO) by αAc, αBc, and αABc as a function of αAc, αBc, and αABc concentrations, respectively, for the Chol/MHLL membranes. The MSO for Chol/MHLL membranes at mixing ratios of 0 (0 mol% Chol) and 0.5 (33 mol% Chol) initially increased with increased αAc, αBc, and αABc concentrations. However, the MSO became constant above certain αAc, αBc, and αABc concentrations, suggesting that the binding of αAc, αBc, and αABc with these membranes is saturable. The maximum percentage of membrane surface occupied (MMSO) (i.e., MSO values at binding saturation) by αAc, αBc, and αABc to Chol/MHLL membranes at a mixing ratio of 0 followed the trends: MMSO (αAc) > MMSO (αBc) ≈ MMSO (αABc), with statistical significance (*p* ≤ 0.05) indicating that a higher amount of αAc binds to these membranes compared to αBc and αABc. Similarly, the MMSO by αAc, αBc, and αABc to Chol/MHLL membranes at a mixing ratio of 0.5 followed the trends: MMSO (αAc) > MMSO (αABc) > MMSO (αBc), with statistical significance (*p* ≤ 0.05) indicating that a higher amount of αAc and the least amount of αBc bind to these membranes. Additionally, with an increase in the membrane Chol concentration, the MMSO by αAc, αBc, and αABc significantly decreased (*p* ≤ 0.05), indicating that Chol and CBDs inhibits the binding of αAc, αBc, and αABc to Chol/MHLL membranes.

**Figure 2:**
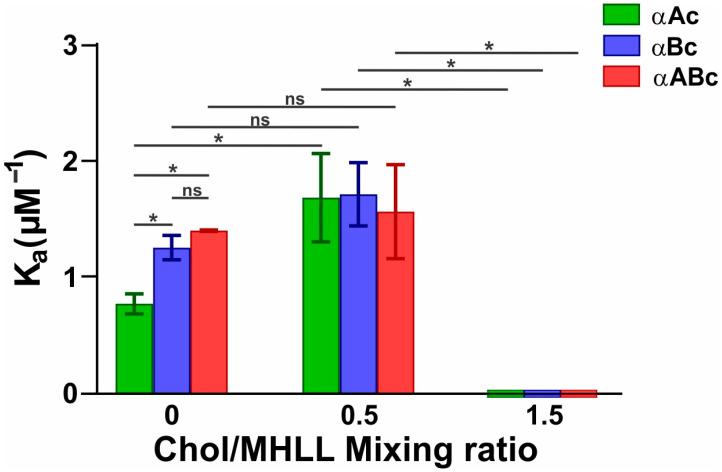
Binding affinity (K_a_) of αAc (green), αBc (blue), and αABc (red) to the Chol/MHLL membranes at mixing ratios of 0, 0.5, and 1.5. As depicted on the left of the x-axis, at a mixing ratio of 0, αAc had the lowest K_a_, followed by αBc, and αABc showed the highest K_a_ to the Chol/MHLL membrane. Shown in the middle of the x-axis, at a mixing ratio of 0.5, the K_a_ values of αAc, αBc, and αABc were all similar and showed the highest K_a_ to the Chol/MHLL membrane. Only the increase in K_a_ of αAc with the increase in Chol content from a Chol/MHLL mixing ratio of 0 to 0.5 was statistically significant (*p* ≤ 0.05); however, the increase in Ka of αBc and αABc with the increase in Chol content from a Chol/MHLL mixing ratio of 0 to 0.5 was not statistically significant. Lastly, as displayed on the rightmost of the x-axis, the binding affinity of αAc, αBc, and αABc at a mixing ratio of 1.5 had no binding and had a K_a_ value of 0 for the Chol/MHLL membrane. The results are the mean ± standard deviation (σ) from at least three independent experiments. * Represents *p* ≤ 0.05, and “ns” represents not significant.

Shown in Figure 2 is the K_a_ of αAc, αBc, and αABc to Chol/MHLL membranes at mixing ratios of 0, 0.5, and 1.5. The K_a_ of αAc, αBc, and αABc to Chol/MHLL membranes at a mixing ratio of 0 followed the trend: K_a_ (αBc) ≈ K_a_ (αABc) > K_a_ (αAc), indicating that the strength of αBc and αABc binding to the membranes was greater than that of αAc. Additionally, the K_a_ values of αAc, αBc, and αABc to Chol/MHLL membranes at a mixing ratio of 0.5 were comparable, i.e., K_a_ (αAc) ≈ K_a_ (αBc) ≈ K_a_ (αABc), indicating that the strength of binding of αAc, αABc, and αABc to the membranes with 33 mol% Chol was similar. The K_a_ of αAc, αBc, and αABc to Chol/MHLL membranes all increased from the Chol-free controls to the samples with 33 mol% Chol, indicating the reduced membrane binding found with increased Chol allows for a faster binding saturation, in turn increasing the relative K_a_; however, the increase in K_a_ of αAc was the only increase that was statistically significant (*p* ≤ 0.05). Furthermore, in high Chol-containing membranes, i.e., at a Chol/MHLL mixing ratio of 1.5, the K_a_ values of αAc, αBc, and αABc to the Chol/MHLL membranes were zero, showing that there was no membrane binding of αAc, αBc, and αABc.

### 2.2. Mobility near the Headgroup Region of Chol/MHLL Membranes with αAc, αBc, and αABc Binding

**Figure 3:**
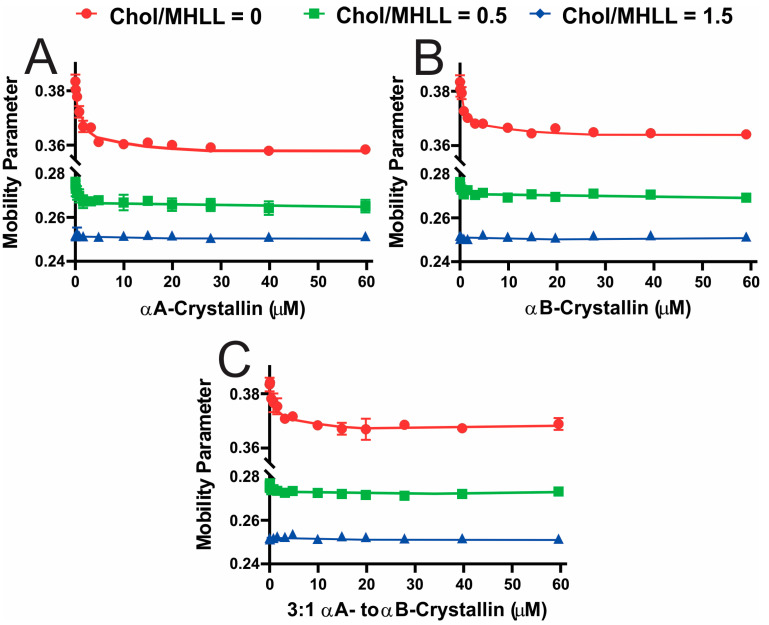
The mobility parameter (h_+_/h_0_) profiles obtained at 37 °C for Chol/MHLL membranes at different mixing ratios plotted as a function of αA-crystallin (αAc), αB-crystallin (αBc), and 3:1 αA- to αB-crystallin (αABc) concentrations. (**A**–**C**) Mobility parameter profiles for Chol/MHLL membranes with mixing ratios of 0 (0 mol% Chol), 0.5 (33 mol% Chol), and 1.5 (60 mol% Chol) with binding of αAc, αBc, and αABc, respectively. These graphs show a significant (*p *≤ 0.05) decrease in mobility near the membrane headgroup region with adding Chol in the membranes. Additionally, at mixing ratios of 0 and 0.5, there was a significant (*p *≤ 0.05) decrease in overall membrane mobility near the headgroup region with αAc, αBc, and αABc membrane binding, except for αABc binding to Chol/MHLL membranes at a mixing ratio of 0.5, where the overall decrease in mobility was not statistically significant at *p *≤ 0.05. There was no significant change in mobility with protein binding at a mixing ratio of 1.5. The error bars were calculated from the average of three independent experiments. Some error bars are small and not visible in the figure.

Figure 3A–C display the mobility parameter, a measure of membrane mobility near the headgroup region, as a function of αAc, αBc, and αABc concentrations, respectively, for Chol/MHLL membranes. As seen in Figure 3A–C, the mobility parameter for the Chol/MHLL membranes with mixing ratios of 0 and 0.5 decreased with an increase in αAc, αBc, and αABc concentrations, indicating that with membrane binding, the membrane regions near the headgroup become less mobile. The overall decrease in the mobility parameter with αAc, αBc, and αABc binding was statistically significant at *p* ≤ 0.05 for the Chol/MHLL membranes at mixing ratios of 0 and 0.5, except for αABc binding with Chol/MHLL membranes at a mixing ratio of 0.5, where the overall decrease was not statistically significant at *p* ≤ 0.05. At Chol/MHLL mixing ratios of both 0 and 0.5, the overall decrease in the mobility parameter was highest when αAc bound to these membranes compared to αBc and αABc because the MMSO by αAc was highest compared to αBc and αABc. This result, therefore, indicates that the higher the MMSO, the larger the overall decrease in mobility, and vice versa. However, this decrease in the mobility parameter with an increase in αAc, αBc, and αABc concentrations was less pronounced with an increase in the Chol concentration in the Chol/MHLL membranes. Moreover, at mixing ratios of 0 and 0.5 in the absence of αAc, αBc, or αABc, the membranes’ mobility parameter decreased with each increase in Chol concentration, meaning there was a significant decrease (*p* ≤ 0.05) in mobility from 0 mol% to 33 mol% Chol and from 33 mol% to 60 mol% Chol. Therefore, as less αAc, αBc, and αABc bound with the membrane with an increase in the Chol concentration, the ability of αAc, αBc, and αABc to decrease the mobility near the headgroup regions was reduced,, and diminished at a high Chol content.

### 2.3. Order near the Headgroup Region of Chol/MHLL Membranes with αAc, αBc, and αABc Binding

**Figure 4:**
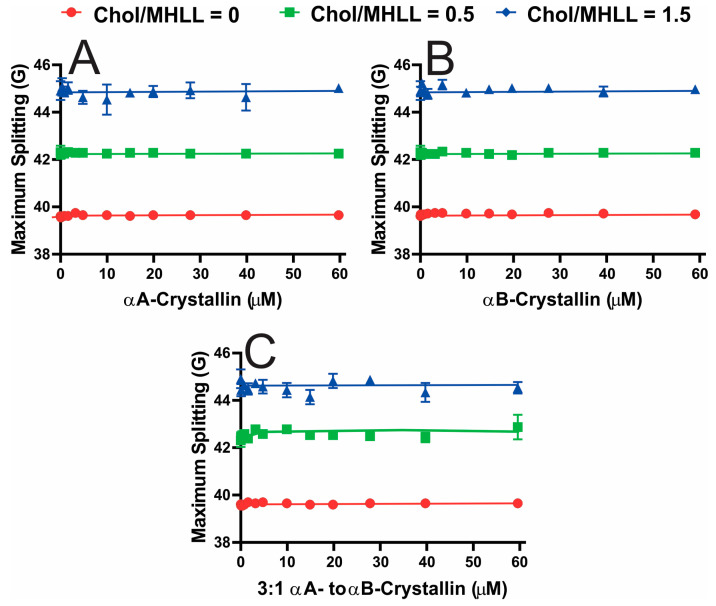
The maximum splitting profiles obtained at 37 °C for the Chol/MHLL membranes at different mixings ratios plotted as a function of αA-crystallin (αAc), αB-crystallin (αBc), and 3:1 αA- to αB-crystallin (αABc) concentrations. (**A**–**C**) Maximum splitting profiles for αAc, αBc, and αABc binding to Chol/MHLL membranes, respectively. As depicted, the maximum splitting of Chol/MHLL membranes significantly (*p* ≤ 0.05) increased with increased Chol content but was not significantly impacted by αAc, αBc, or αABc membrane binding. The error bars were calculated from the average of three independent experiments.

Figure 4A–C display the maximum splitting profiles as a function of αAc, αBc, and αABc concentrations, respectively, for Chol/MHLL membranes. The maximum splitting provides information about the order near the headgroup region when αAc, αBc, and αABc bind the membrane. No significant change (*p* ≤ 0.05) in the maximum splitting with an increase in αAc, αBc, and αABc concentration was observed for Chol/MHLL membranes with mixing ratios of 0, 0.5, and 1.5, except a slight, but non-significant (*p* ≤ 0.05), overall increase in maximum splitting was observed when αABc bound the Chol/MHLL membranes at a mixing ratio of 0.5. Additionally, with each increase in the membrane Chol concentration, there were significant increases (*p* ≤ 0.05) in maximum splitting values, indicating that the addition of Chol and formation of CBDs significantly increased the membrane order near the headgroup region.

### 2.4. Hydrophobicity near the Headgroup Region of Chol/MHLL Membranes with αAc, αBc, and αABc Binding

**Figure 5:**
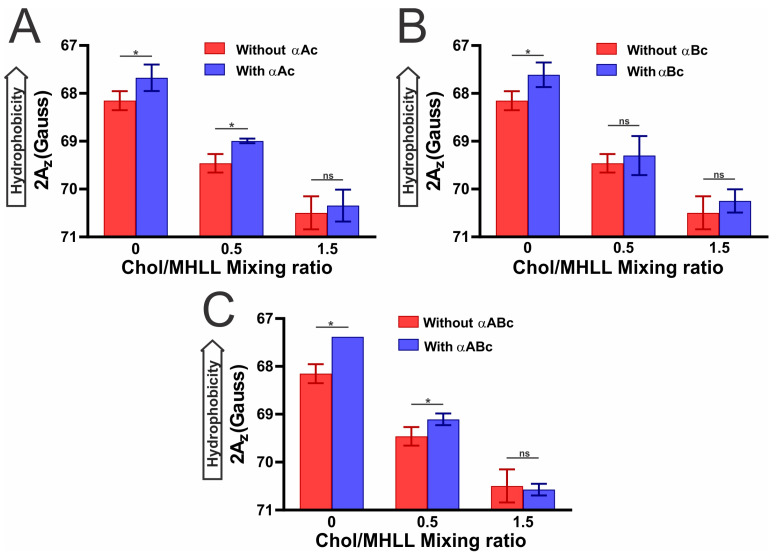
Hydrophobicity near the surface of Chol/MHLL membranes at Chol/MHLL mixing ratios of 0 (0 mol% Chol), 0.5 (33 mol% Chol), and 1.5 (60 mol% Chol), measured (**A**) with ~60 μM αA-crystallin (αAc) and without αAc, (**B**) with ~60 μM αB-crystallin (αBc) and without αBc, and (**C**) with ~60 μM 3:1 αA- to αB-crystallin (αABc) and without αABc. A decrease in 2 Az indicates an increase in hydrophobicity, where Az is the z-component of the hyperfine interaction tensor. The EPR measurements were performed at −165 °C. The error bars were calculated from the average of three independent experiments. * Represents *p* ≤ 0.05, and “ns” represents not significant.

Figure 5 shows the hydrophobicity near the headgroup regions of the Chol/MHLL membranes at mixing ratios of 0, 0.5, and 1.5 measured with and without (A) αAc, (B) αBc, and (C) αABc. For Chol/MHLL membranes at a mixing ratio of 0, hydrophobicity near the headgroup region of the membrane significantly increased (*p* ≤ 0.05) when αAc, αBc, and αABc bound to the membrane. The binding of αAc and αABc to Chol/MHLL membranes with a mixing ratio of 0.5 significantly increased (*p* ≤ 0.05) the hydrophobicity near the membrane surface, whereas the binding of αBc to these membranes resulted in no significant change in the hydrophobicity near the membrane surface. For Chol/MHLL membranes at a mixing ratio of 1.5, no significant difference in the hydrophobicity was observed near the membrane surface in the presence of αAc, αBc, and αABc. The hydrophobicity near the headgroup region of the Chol/MHLL membrane decreased with an increase in the Chol concentration due to Chol moving the polar headgroups apart, resulting in increased water penetration near the surface of these membranes. The decreased hydrophobicity near the surface of Chol/MHLL membranes with increased Chol content (Figure 5) and the decreased MMSO by αAc, αBc, and αABc with increased Chol content (Figure 1) indicated that αAc, αBc, and αABc binding to the membrane is modulated by the surface hydrophobicity of these membranes, suggesting hydrophobic interaction of αAc, αBc, and αABc with membranes.

**Figure 6:**
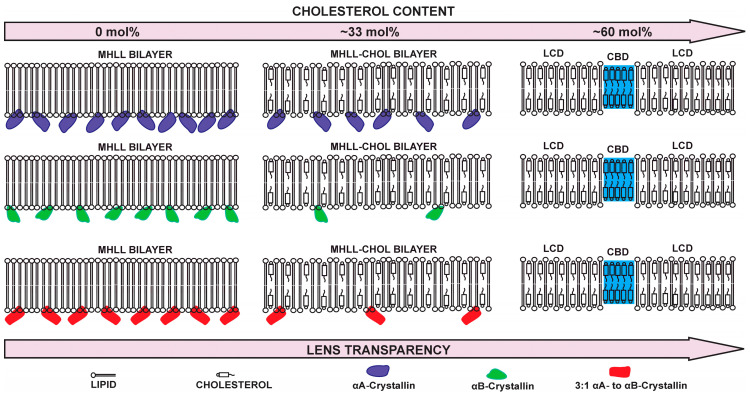
Schematic displaying the binding of αAc, αBc, and αABc to the Chol/MHLL membranes with increasing Chol content. With an increase in Chol content, displayed from left to right, the membrane became saturated with Chol, and a lipid cholesterol domain (LCD) formed. Moreover, as depicted by the blue highlighted regions, with a further increase in Chol content, cholesterol bilayer domains (CBDs) started to form above ~46 mol% Chol within Chol/MHLL membranes, preventing the membrane binding of αAc, αBc, and αABc. CBD is represented by blue, and αAc, αBc, and αABc oligomers are represented by violet, green, and red colors respectively. The actual sizes of αAc, αBc, and αABc oligomers represented in Figure 6 are much larger than shown in the schematic display.

Based on our results in Figure 1, we have drawn a schematic showing the binding of αAc, αBc, and αABc to Chol/MHLL membranes with an increasing Chol content, as illustrated in Figure 6.

## 3. Discussion

The results from this study showed that αAc, αBc, and αABc can all bind to the Chol/MHLL membranes. As depicted in Figure 6, αAc was shown to bind the most to the Chol/MHLL membranes and had the highest MMSO compared to that found for αBc and αABc, which displayed relatively similar levels of membrane binding. The differences in the MMSO by αAc, αBc, and αABc may result from the differences in the oligomeric size, polydispersity, and surface hydrophobicity of αAc, αBc, and αABc. As αAc has the highest MMSO at both mixing ratios of 0 and 0.5 and the highest level of polydispersity [52,53], it may form a higher number of smaller oligomers that are able to more effectively bind below the surface of the membrane, allowing for increased binding. We determined the hydrodynamic radius (R_h_) and percentage of polydispersity (% Pd) by dynamic light scattering (DLS) for αAc, αBc, and αABc. We found that the R_h_ values for αAc, αBc, and αABc were 8.18 ± 0.48 nm, 7.73 ± 0.13 nm, and 8.04 ± 0.38 nm, respectively, with the increased R_h_ indicating a larger size of oligomers, which is in agreeance with a previously reported R_h_ for αAc, αBc, and αABc [52,54]. Additionally, the %Pd values estimated for αAc, αBc, and αABc were 15.74 ± 5.03%, 5.65 ± 2.34%, and 7.38 ± 3.02%, respectively. A smaller %Pd value indicates that there is a reduced distribution of oligomer size, while a larger %Pd value indicates that there is an increased oligomer size distribution. Therefore, αAc had the largest variation in oligomer size, with αABc showing intermediate variation and αBc having the least variation in size. In agreeance with the %Pd values, previous studies have found that αBc has less polydispersity and primarily forms 24-subunit oligomers, while αAc and αABc can form 24-mers but are generally more heterogeneous [52,53]. This increased level of polydispersity for αAc may lead to the formation of smaller oligomers that more easily bind between the membranes’ headgroup region, in turn allowing for increased membrane binding. Moreover, the binding of smaller oligomers may occupy less space on the membrane, allowing for an increased number of oligomers to bind to the membrane surface. In agreeance with this idea, Tiondro et al. found that αAc insertion into the membrane depends on the oligomer size of αAc [83]; however, further research is still needed along this line. This increased polydispersity may, therefore, be a primary reason that αAc was shown to have the highest possible MMSO compared to αBc and αABc. Interestingly, while αABc had more polydispersity than αBc, αABc showed similar binding levels to those of αBc. This variation in binding may be due to the quaternary structure of the αABc oligomers resulting in less exposed hydrophobic residues, or the relative amount of small oligomer complexes may be reduced relative to that found for αAc, in turn allowing for increased αAc membrane binding and explaining the similar binding levels of αBc and αABc.

In addition to the protein size and structure, the binding of αAc, αBc, and αABc was strongly modulated by the Chol content of the MHLL membranes, with an increase in Chol causing an increase in binding inhibition. The Figure 6 schematic showing the MHLL bilayer (0 mol% Chol), MHLL-Chol bilayer (33 mol% Chol), and CBD coexisting with LCD (60 mol% Chol) was drawn based on our earlier studies [63,67,79,84]. As mentioned in our earlier studies [63,67,79,84], with an increase in Chol content in the membranes, membranes are saturated with Chol, and the lipid cholesterol domain (LCD) is formed (in our previous papers, we used phospholipid cholesterol domain (PCD) to represent the membranes saturated with Chol [63,67,84]; here, we used LCD to represent the membrane saturated with Chol to emphasize that lipids include both phospholipids and sphingolipids). With further increases in the Chol concentration in Chol/MHLL membranes, CBDs start to form above 46 mol% Chol [67], and CBDs coexist with LCD, as shown in Figure 6. Our previous studies demonstrated that CBDs begin to form at 48, 46, 50, and 33 mol% Chol in the SM, POPS, POPC, and POPE bilayers, respectively [79]. The assumption used to estimate the formation of CBDs within the Chol/MHLL membranes was the weighted sum of the individual Chol content values for each lipid (POPC, POPE, POPS, and SM), with the weight equal to the mol% of each lipid in the membrane, as described by us previously [67,79]. As displayed in Figure 6, at a mixing ratio of 0.5, the binding of αAc, αBc, and αABc were all significantly reduced, with the binding of αAc being the least inhibited compared to αBc and αABc. Specifically, at a mixing ratio of 0.5, the MMSO by αAc, αBc, and αABc decreased by approximately 40%, 71%, and 61%, respectively, resulting in αAc having approximately double the MMSO of the Chol/MHLL membrane than that seen for αBc and αABc. These results indicated that 33 mol% Chol in the Chol/MHLL membrane was the least impactful in decreasing the amount of αAc binding compared to that of αBc and αABc. As further shown in Figure 6, at a mixing ratio of 1.5 (60 mol% Chol), the membrane became saturated with Chol, leading to the formation of LCD and CBDs, resulting in a complete inhibition of αAc, αBc, and αABc binding [63,67]. Therefore, while at a reduced Chol content αAc exhibited the highest amount of membrane binding compared to αBc and αABc, with a high Chol content where CBDs are formed within the membranes, the reconstituted 3:1 heteromeric complex of αAc to αBc (i.e., αABc) and any of the αABc, αAc, and αBc oligomeric states were not able to bind the Chol/MHLL membrane. The increased binding of αAc in the presence of Chol is again likely due to the increased level of polydispersity resulting in increased numbers of smaller oligomers that are able to still bind near the membrane headgroup region in the reduced membrane space, seen with the addition of Chol. Another possible factor impacting the interactions of αAc, αBc, and αABc is the development of lipid rafts or phase-separated microdomains. Lipid rafts are generally formed in the liquid-ordered (*l*_o_) [85,86,87] phase of membranes typically rich in sphingolipids and Chol [88]. At 37 °C, the phase diagram for the Chol/PC membrane system showed that the liquid-disordered (*l*_d_) phase plus *l*_o_ phase formed with a Chol content between ~8 mol% and ~28 mol%, but existed solely in the *l*_o_ phase above 30 mol% Chol [89], while the Chol/SM existed in the *l*_o_ phase after ~30 mol% Chol [90]. Moreover, at 37 °C, both the Chol/PC and Chol/SM membranes existed in the *l*_d_ phase at 0 mol% Chol [89,90]. Based on these observations, the investigated Chol/MHLL membranes, rich in SM with 33 mol% Chol, might contain raft domains primarily in the *l*_o_ phase that reduce the MMSO by αAc, αBc, and αABc.

As discussed above, the MMSO by αAc was the largest, followed by αABc and αBc. However, interestingly, the K_a_ trend was the opposite of that reported for the MMSO by αAc, αBc, and αABc to the Chol-free Chol/MHLL membranes. The K_a_ of αAc, αBc, and αABc to the Chol/MHLL membranes at a lipid/Chol mixing ratio of 0 followed the trend: K_a_ (αBc) ≈ K_a_ (αABc) > K_a_ (αAc), while at a mixing ratio of 0.5, the K_a_ between all three was relatively similar. Therefore, in the absence of Chol, the binding of αBc and αABc to the MHLL membranes was stronger than that of αAc. These K_a_ results suggested that Chol modulates the strength of αAc, αBc, and αABc binding to membranes, with an increase in Chol reducing the total binding, allowing for similar binding strengths and bringing the K_a_ values between all three to similar values. At a higher Chol content (i.e., with 60 mol% of Chol), CBD coexists with LCD, as shown in Figure 6, where both MMSO by αAc, αBc, and αABc and K_a_ values of αAc, αBc, and αABc were zero, indicating the complete inhibition of αAc, αBc, and αABc binding to the Chol/MHLL membranes. These results showed that irrespective of αAc, αBc, and αABc concentrations, the MMSO and K_a_ became zero at saturating Chol content, where CBDs formed within the membranes. This effect of Chol and CBDs on the binding of αAc, αBc, and αABc is important, as the Chol content and amount and size of CBDs in a transparent human lens membrane increases with aging [71], but the development of cataracts leads to a reduction in the lens Chol content and a decrease in the amount and size of CBDs [76,91,92]. Therefore, these results suggest that Chol and CBDs in the lens membrane inhibit αAc, αBc, and αABc binding with the membrane and likely increase the concentration of water-soluble αAc, αBc, and αABc in the lens cytoplasm, favoring its chaperone function, maintaining lens cytoplasm homeostasis, and ultimately preventing cataract formation. Consequentially, the reduction of Chol and CBD content in cataractous lenses likely promotes α-crystallin binding and the formation of HMW protein aggregates, ultimately promoting the development and progression of cataracts.

The binding of α-crystallin (αAc, αABc, and αABc) and the Chol content of the Chol/MHLL membrane were both shown to alter the physical properties of membranes. With the binding of αAc, αBc, and αABc, there was a decrease in membrane mobility near the headgroup region, with the highest MMSO by αAc resulting in the largest decreases in mobility. Relatedly, with the addition of Chol at a mixing ratio of 0.5, the loss of mobility seen with αAc, αBc, and αABc binding was reduced due to the inhibition of αAc, αABc, and αABc membrane binding. This trend was further exacerbated at a mixing ratio of 1.5, where, with the loss of binding, there was no clear change in mobility. These data indicate that the binding of αAc, αBc, and αABc decreased mobility near the membrane headgroup region, with increased binding directly correlating with decreased mobility. Additionally, in the protein-free controls, the addition of the Chol was shown to directly reduce the mobility of the membrane near the headgroup region. In combination, the two observed effects implied that an increase in the membrane Chol concentration decreased the membranes’ mobility near the headgroup regions and inhibited the ability of αAc, αBc, and αABc to decrease mobility near the headgroup regions through membrane binding. Furthermore, the antagonization capacity of Chol to diminish membrane binding, resulting in a decrease in mobility, was larger for αBc and αABc than αAc. Previously, we observed a similar decrease in the mobility parameter of individual and two-component lipid membranes, cholesterol-containing lipid membranes, and a model of the lens-lipid membranes after native bovine lens αABc binding [63,64,67,68]. In addition to membrane mobility, the order of the membrane near the headgroup region was shown to be strongly modulated by the addition of Chol but was not significantly altered with αAc, αABc, and αABc binding. In the absence of αAc, αABc, and αABc, the progressive addition of Chol resulted in significant increases in membrane order, with the Chol/MHLL membrane at a mixing ratio of 0 having the lowest maximum splitting values, while the highest values were found in the membranes with a mixing ratio of 1.5. Therefore, with the addition of Chol and the formation of CBDs at a high Chol content, the membrane became increasingly ordered near the headgroup region but was not strongly affected by αAc, αABc, and αABc binding. In agreeance, in our previous studies on native bovine lens αABc interactions with one and two-component lipid membranes, Chol-containing membranes, and model lens-lipid membranes, we found that there was generally no significant change in maximum splitting with an increased αABc concentration [63,64,67,68].

The results presented in this paper further showed that αAc, αBc, and αABc membrane binding caused an increase in hydrophobicity near the membrane surface, indicating that αAc, αBc, and αABc binding is performed through hydrophobic interactions. Previous studies also reported the hydrophobic interaction of αABc with the membranes [26,45,67,83]. In the absence of Chol, the binding of αAc, αBc, and αABc resulted in significant increases in hydrophobicity, forming a hydrophobic barrier on the membrane surface, and such a hydrophobic barrier likely creates a barrier for the passage of ionic and polar molecules, including antioxidants (glutathione), creating the oxidative environment inside the lens that leads to cataract development. It is very likely that when the hydrophobic residues at the surface of αAc, αBc, and αABc binds the lens membrane, the water molecules around the polar headgroup regions are expelled, increasing the hydrophobicity (decrease in polarity). However, with the increase in Chol content in the membranes (i.e., Chol/MHLL mixing ratio of 0.5), the binding of αAc, αBc, and αABc to the membrane was reduced, resulting in a decrease in the increase in hydrophobicity with αAc, αBc, and αABc binding. With the further increase in Chol content in the membrane (i.e., Chol/MHLL mixing ratio of 1.5) where CBDs are formed, the binding of αAc, αBc, and αABc to the membrane was completely diminished, suggesting that high Chol and CBDs inhibit the binding of αAc, αBc, and αABc to the membrane, preventing the formation of such hydrophobic barriers and protecting against cataract formation. Moreover, in the absence of αAc, αBc, and αABc, the addition of Chol resulted in significant decreases in hydrophobicity, with the largest amount of Chol at a mixing ratio of 1.5 causing the most substantial decrease in hydrophobicity. Therefore, the addition of Chol in the Chol/MHLL membrane and formation of CBDs at a high Chol content likely separates the polar headgroups, which increases water penetration near the membrane surface [63,67] and, consequently, reduces the hydrophobicity around the membrane headgroup region, preventing protein binding. Our study suggested that with increased Chol content, there is a decrease in hydrophobicity and a corresponding decrease in the binding of α-crystallin to Chol/MHLL membranes, suggesting that the lower the hydrophobicity at the membrane surface, the lower the binding of α-crystallin to the membrane. In the cataractous lens cortical and nuclear membrane, there is less Chol compared to age-matched transparent lens cortical and nuclear membranes [76,91,92]; therefore, the development of a strategy to increase Chol content in the lens membrane may reduce α-crystallin binding and, consequently, prevent the development and progression of cataracts. Furthermore, developing cholesterol derivative compounds that significantly decrease the hydrophobicity on the lens membrane surface would reduce α-crystallin binding to the membrane and likely prevent the development and progression of cataracts.

The results presented in this study are in agreeance with our previous work on native bovine lens αABc membrane binding. From our past studies, we have found that native bovine lens αABc binds the individual lipid membranes composing the eye lens membrane [64], model of eye lens-lipid membranes [67,68], and isolated cortical and nuclear lens-lipid membranes from single bovine lenses. Moreover, in all of these studies, we have found that αABc membrane binding is performed via hydrophobic interactions and is strongly modulated by the lens-lipid and Chol composition [63,64,67,68]. Relatedly, in this study, the addition of Chol and the formation of CBDs were consistently shown to lower the hydrophobicity and, consequently, reduce the binding of αAc, αBc, and αABc with the membrane. Therefore, it appears that αAc, αBc, and αABc follow this trend and can bind to the eye lens-lipid membrane through hydrophobic interactions, with the addition of Chol into the Chol/MHLL membrane reducing the hydrophobicity near the headgroup region and ultimately diminishing membrane binding and preventing the formation of the hydrophobic barrier on the membrane surface, and likely maintaining lens homeostasis and lens transparency. The results reported in this manuscript are significant, as they characterize the nature of the interactions of αAc, αBc, and αABc with the Chol/MHLL membranes, the effects of binding on the physical properties (mobility, order, and hydrophobicity) of membranes near the headgroup regions, and the inhibitory role of Chol and CBDs on αAc, αBc, and αABc membrane interactions.

## 4. Materials and Methods

### 4.1. Materials

Cholesterol (Chol), sphingomyelin (SM), and phospholipids (PLs): 1-palmitoyl-2-oleoyl-sn-glycero-3-phosphatidylCholine (POPC), 1-palmditoyl-2-oleoyl-sn-glycero-3-phosphoethanolamine (POPE), and 1-palmitoyl-2-oleoyl-sn-glycero-3-phosphatidylserine (POPS), were obtained dissolved in chloroform from Avanti Polar Lipids, Inc. (Alabaster, AL, USA). Cholesterol analog cholestane spin label (CSL), HEPES, Tris-HCl, NaN_3_, sodium chloride (NaCl) lysozyme, deoxycholic acid, and DNase I were obtained from Sigma Aldrich (St. Louis, MO, USA). Isopropyl-1-thio-β-D-galactopyranoside (IPTG), ampicillin, phenylmethylsulfonyl fluoride, and polyethyleneimine were obtained from Santa Cruz Biotechnology, Inc. (Dallas, TX, USA). Recombinant human αAc and αBc were expressed and purified, and the reconstituted 3:1 heteromeric complex of αAc to αBc (i.e., αABc) was prepared using the methods described in Section 4.2 and stored in HEPES buffer (10 mM HEPES, 100 mM NaCl, pH = 7.4). All preparations of α-crystallin (αAc, αBc, and αABc) and the Chol/MHLL membranes, as well as associated binding studies, were performed in HEPES buffer (10 mM HEPES, 100 mM NaCl, pH = 7.4).

### 4.2. Expression and Purification of Recombinant Human αAc and αBc

The pET-43.1a(+) plasmids containing the genes for human αAc and αBc were obtained from Genescript USA Inc. The plasmids containing human αAc and αBc genes were transferred to competent *E*. *coli* BL21 (DE3) cells by the heat shock method, and the cells were then spread in an LB agar plate containing 100 µg/mL of ampicillin, and single colonies were obtained following the manufacturer’s protocol (Thermo Scientific user guide, Pub. No. MAN0018595, Rev. A.0 [93]). The expression and purification of αAc and αBc were performed using the methodology previously described in [94]. Briefly, the culture was induced with 0.5 mM of IPTG after reaching the optical density of ~0.5 at 600 nm and further incubated at 37 °C for ~3 h at 225 rpm. Cells were harvested by centrifugation at 5000× *g* for 10 min at 4 °C. Cell pellets were resuspended in ice-cold lysis buffer (50 mM Tris-HCl, 100 mM NaCl, pH 8.0) containing 130 µM of phenylmethylsulfonyl fluoride and 260 µg/mL of lysozyme. After incubation for 20 min in ice, 4 mg of deoxycholic acid per gram of original pellet was added, followed by vigorous shaking for ~30 min at 37 °C. The viscous lysate was added with 10 µL of DNase I (2 mg/mL stock) per gram of original pellet and placed at room temperature until no longer viscous (~30 min). The cell debris was removed by centrifugation at 17,000× *g* for 15 min at 4 °C, and the supernatant was further centrifuged at 65,000× *g* for 30 min at 4 °C. The supernatant was then treated with dithiothreitol (10 mM final concentration) and polyethyleneimine (0.12% final concentration) and incubated at room temperature for 10 min, followed by centrifugation at 17,000× *g* for 10 min at 4 °C. The supernatant was dialyzed overnight in 20 mM of Tris buffer, pH 8.5, and filtered with a 0.22 µm syringe filter before loading in the AKTA go protein purification system. Recombinant human αAc and αBc were purified from lysates following a two-step chromatography: anion-exchange (HiPrep 16/10 DEAE FF column in 20 mM Tris buffer, pH 8.5, 0–1 M NaCl gradient) and size-exclusion (Hiload 16/600 Superose 6 pg gel filtration column in 10 mM HEPES, 150 mM NaCl, pH 7.4). Purified human αAc and αBc were concentrated using Amicon Ultra-15 filters by centrifuging at 5000 rpm at 4 °C and dialyzed in buffer A (10 mM HEPES, 100 mM NaCl, pH 7.4) at 4 °C. The purity of αAc and αBc was confirmed by sodium dodecyl sulfate–polyacrylamide gel electrophoresis (SDS-PAGE). The concentrations of αAc and αBc were determined in triplicate by measuring the UV absorbance at 280 nm using extinction coefficients of 14,502 M^−1^ cm^−1^ and 13,980 M^−1^ cm^−1^, respectively, and molecular weights of 19.91 kDa and 20.16 KDa, respectively. The extinction coefficients and molecular weights for αAc and αBc were estimated using the ProtParam tool on the Expasy server [95]. The αABc heteropolymer formed with a 3:1 ratio of αAc to αBc was made by combining αAc and αBc in the appropriate molar ratio and incubating them at 37 °C for 24 h in buffer A [26]. The αAc, αBc, and αABc were used for EPR measurements.

### 4.3. EPR Sample Preparation and Measurements

The four major eye lens lipids (SM and three PLs), i.e., 66% SM, 11% POPC, 8% POPS, and 15% POPE, were used to mimic the lipid composition of a 60-year-old MHLL membrane [67,96] and increasing Chol concentrations were used to mimic the increase in lens Chol content seen with aging [71]. The lipid composition of 60-year-old MHLL membranes was acquired from a previous study by Deeley et al. [96], where they used mass spectrometry to find the lipid composition of the 60-year-old human lenses from three 60-year-old females and one 60-year-old male to determine the eye lens-lipid composition of a 60-year-old human [96]. For the preparation of the Chol/MHLL membranes at mixing ratios of 0, 0.5, and 1.5, small unilamellar vesicles (SUVs) with 1 mol% CSL spin label were prepared using the rapid solvent exchange method, followed by probe-tip sonication, as described in our previous work [67]. The use of a Chol/MHLL membrane in the proposed research enabled us to manipulate the Chol content within the membrane in a controlled manner, allowing us to know the role of increased Chol content in αAc, αBc, and αAbc membrane binding. The Chol/MHLL membranes were mixed with varying concentrations of αAc, αBc, or αABc to a total volume of 70 µL and incubated in a Corning benchtop incubator (Corning, NY, USA) for 16 h at 37 °C with gentle shaking. The concentration of lipids plus Chol was maintained at 11.4 mM, and αAc, αBc, and αABc were varied from 0 to ~60 μM. The incubated samples were loaded into a 1.0 mm i.d. gas-permeable methylpentene polymer (TPX) capillary for EPR measurements at 37 °C and at about −165 °C using an X-band EPR spectrometer connected with the temperature-control accessories. All experiments on the binding of αAc, αBc, and αABc with the Chol/MHLL membranes at mixing ratios of 0, 0.5, and 1.5 were repeated at least from three samples prepared independently, and statistical analysis (Student’s *t*-test) was performed. The concentration of lipids plus Chol, αAc, αBc, and αABc, and the experimental conditions were chosen based on our previous studies [63,67,68]. The EPR spin-labeling method developed in our laboratory [23,63,64,67,68] was used to measure the MSO by αAc, αBc, and αABc and K_a_ values of αAc, αBc, and αABc to Chol/MHLL membranes. The EPR approach has the unique ability to simultaneously provide information on the binding of αAc, αBc, and αABc to membranes (i.e., MSO by αAc, αBc, and αABc and the K_a_ values of αAc, αBc, and αABc to the membranes) and the physical properties (mobility parameter, maximum splitting, and hydrophobicity) of the membranes with αAc, αBc, and αABc binding.

**Figure 7:**
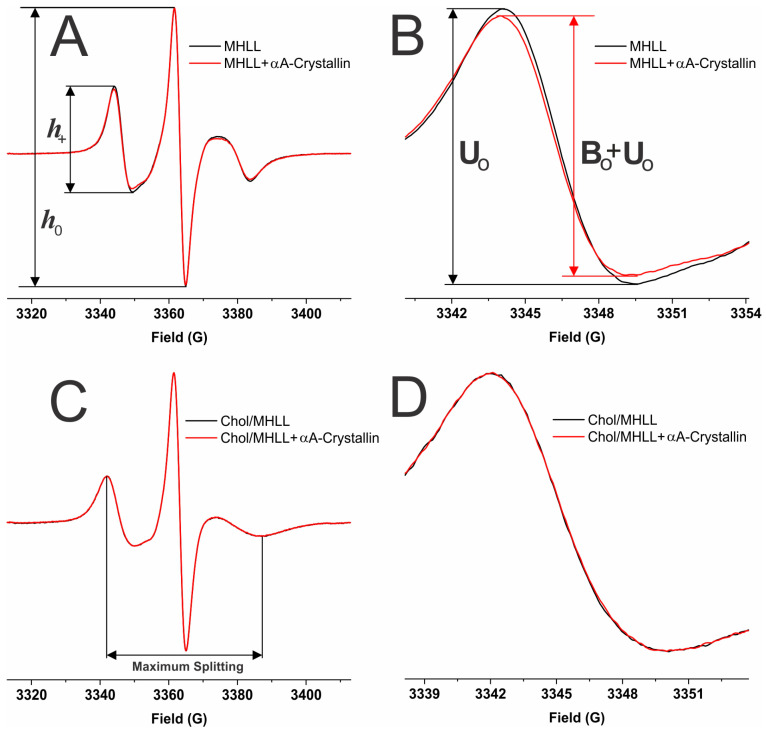
(**A**) Representative EPR spectra of CSL in MHLL membranes with a Chol/MHLL mixing ratio of 0 (no Chol) in the absence of αA-crystallin (αAc) and 59.7 μM αAc shown in black and red, respectively. (**C**) Representative EPR spectra of CSL in a Chol/MHLL membrane with a Chol/MHLL mixing ratio of 1.5 in the absence of αAc (black) and 59.7 μM αAC (red). Cholesterol bilayer domains (CBDs) were formed at a mixing ratio of 1.5 [67]. (**B**,**D**) Zoomed low-field line of the spectra in (**A**) and (**C**), respectively. EPR measurements were taken at 37 °C. As shown in (**A**), the ratio of peak-to-peak intensity of the low-field line (h_+_) and the central line (h_0_) of the EPR spectra was used to calculate the mobility parameter (h_+_/h_0_) of the membranes. (**B**) The unbound (U_0_) and unbound plus bound (U_0_ + B_0_) contributions used to calculate the percentage of membrane surface occupied (MSO) by αAc and K_a_. The horizontal distance between the low- and high-field lines was used to calculate the maximum splitting (**C**).

Displayed in Figure 7 are demonstrative EPR spectra, taken at 37 °C for the CSL spin label in the Chol/MHLL membrane in the absence (black) and presence of αAc (red). From these spectra, we obtained information on the MSO, K_a_, mobility parameter, and maximum splitting. As displayed in Figure 7A, the ratio of peak-to-peak heights of the low-field line (h_+_) and the central line (h_0_) provided us with data on the mobility parameter. The mobility parameter provides information regarding the mobility near the headgroup regions of membranes with and without protein binding [63,64,97]. Further displayed in Figure 7B is the zoomed-in low-field lines of the EPR spectra displayed in Figure 7A, showing that αAc binding to the Chol/MHLL membrane decreased the peak-to-peak intensity of the low-field line relative to that recorded for the protein-free control membrane. Additionally, shown in Figure 7C are the representative measurements for the maximum splitting value, in which the distance between the peaks of the low-field and high-field lines represents maximum splitting. The maximum splitting provides information regarding the order of the membranes near the headgroup regions of membranes with and without protein binding [63,64,97].

As further depicted in Figure 7B, the low-field line of the EPR spectra from the control membranes without αAc was used as an unbound contribution (U_0_), while the low-field line of the spectra with αAc binding was used as an unbound plus bound (U_0_ + B_0_) contribution. These U_0_ and U_0_ + B_0_ contributions were used to calculate the percentage of CSL spin labels near the surface of the membrane that were affected by αAc binding, as described in our previous studies [63,64]:

(1) $$\%\;\mathrm{CSL}\;\mathrm{spin}\;\mathrm{labels}\;\mathrm{affected}=\left\{\frac{U_{0}-(B_{0}+U_{0})}{U_{0}}\right\}\times 100\%$$

DLS measurements were taken using a DynaPro instrument (Wyatt Technology Corp., Santa Barbara, CA, USA) using regularization methods (Dynamics software, version 7) to find the R_h_ of the SUVs from the experimentally analyzed Chol/MHLL membrane SUVs. Individual R_h_ values were used to calculate the MSO by αAc, αBc, and αABc for each set of Chol/MHLL samples. DLS measurements showed the average R_h_ of the vesicles with a Chol/MHLL mixing ratio of 0 and 0.5 to be ~56 nm and the average R_h_ of the vesicles with a Chol/MHLL mixing ratio of 1.5 to be ~59 nm. With an R_h_ of 56 nm, ~54% of the CSL spin labels were on the outer surface of the Chol/MHLL membranes. As the only spin labels affected by αAc, αBc, and αABc binding were those on the outer surface, the corrected percentage of CSL spin labels affected by αAc, αBc, and αABc binding was estimated by multiplying Equation (1) by the correction factor. A correction factor of 100/54 was used for the Chol/MHLL membranes to evaluate the corrected % CSL spin label affected by αAc, αBc, and αABc binding, ultimately providing us with the MSO by αAc, αBc, and αABc [63,64,67]. Using the MSO values obtained at varying αAc, αBc, and αABc concentrations, the K_a_ values were calculated using the one-site ligand-binding model by fitting MSO values shown in Figure 1A–C. An example calculation for the Chol/MHLL membrane with the correction factor reads as follows:

(2) $$\%\;\mathrm{Membrane}\;\mathrm{surface}\;\mathrm{occupied}\;(\mathrm{MSO})=(\%\;\mathrm{CSL}\;\mathrm{spin}\;\mathrm{labels}\;\mathrm{affected})\times \left(\frac{100}{54}\right)$$

The z-component of the hyperfine interaction tensor (A_z_) for the CSL spin labels in the Chol/MHLL membranes was measured from samples frozen with liquid nitrogen to approximately −165 °C. The horizontal distance between the low-field line and the high-field line of the EPR spectra collected at −165 °C provided us with the 2 A_z_ value [67]. This 2 A_z_ value is a measure of hydrophobicity [67,75,81], in which an increased 2 A_z_ value corresponds to a reduced level of hydrophobicity near the headgroup region of the membrane [67,68].

### 4.4. Statistics

All data acquired for the interactions of αAc, αBc, and αABc with the Chol/MHLL membranes at mixing ratios of 0, 0.5, and 1.5 were expressed as mean and standard deviation from at least three independent experiments. The Student’s *t*-test was used to determine the statistical significance of K_a_, MMSO, mobility parameter, maximum splitting, and hydrophobicity values. For all experiments, a *p*-value ≤ 0.05 was considered statistically significant. 

## Data Availability

Data are contained within the article.
